# Systematic Review of Tibial Stems in Primary Total Knee Arthroplasty

**DOI:** 10.7759/cureus.77563

**Published:** 2025-01-16

**Authors:** Prabu Supramaniam, Arshad Barmare, Siva Chandrasekaran

**Affiliations:** 1 Orthopaedics, Kuala Lumpur Hospital, Kuala Lumpur, MYS; 2 Orthopaedics, Werribee Mercy Hospital, Werribee, AUS; 3 Orthopedic Surgery, St. Vincent's Private Hospital, Werribee, AUS

**Keywords:** aseptic loosening, primary knee arthroplasty, systematic review, tibial stem extension, total knee arthroplasty

## Abstract

Tibial stems are increasingly used in primary total knee arthroplasty (TKA) to enhance stability and reduce component load, particularly in complex cases involving obesity, severe deformity, bone loss, and inflammatory arthritis. However, limited literature exists on their indications, outcomes, and complications. This study systematically reviews the indications for tibial stems in primary TKA, the types of stems used, and their associated outcomes, including revision rates and complications. A systematic review, following Preferred Reporting Items for Systematic Reviews and Meta-Analyses (PRISMA) guidelines, was conducted using PubMed, Embase, and Cochrane databases up to January 2024. Studies comparing stemmed and non-stemmed tibial baseplates, as well as those not comparing with non-stemmed tibial baseplates, were included. Abstracts, commentaries, and case reports were excluded. Twenty-three studies, comprising 2,073 tibial stems (30-145 mm), met the inclusion criteria. The primary indication for tibial stems was in obese patients, with variable body mass index (BMI) thresholds. The use of tibial stems, particularly short cemented stems, demonstrated favorable outcomes in complex primary TKA, including reduced rates of aseptic loosening and improved functional scores. In cases of severe varus deformity and osteoarthritis with associated tibial stress or plateau fractures, tibial stems were effective in reducing complications and improving implant stability. Short cemented tibial stems benefit complex primary TKA, especially in obese patients and those with severe deformities, preserving bone stock, optimizing load transmission, and minimizing complications. Further research is needed to establish standardized guidelines for optimal tibial stem use in primary TKA.

## Introduction and background

In contemporary orthopedic practice, surgeons worldwide utilize a diverse range of total knee implant designs, with some featuring modular components allowing for the attachment of an intramedullary stem extension to either the femoral or tibial components. The primary cause of mechanical implant failure is often associated with issues in the tibial component; thus, tibial stems are commonly favored over femoral stems in complex primary total knee arthroplasties (TKAs) [1]. Tibial stems play a substantial role in improving the mechanical stability of tibial components during TKA procedures and reducing the load on these components [2]. Tibial stems are generally reserved for revision TKAs; however, there have been instances where tibial stems were incorporated in primary TKAs. Hinman et al. conducted a large cohort study comparing 10,476 primary TKA procedures with modular tibial stems to an equal number of matched TKA procedures without tibial stems. Their findings showed that the use of a tibial stem was associated with a reduced risk of revision due to aseptic loosening [3].

The literature on the use of tibial stems in primary TKA is growing, but only a modest number of studies have specifically addressed their indications, outcomes, and potential complications. To the best of our knowledge, we did not find any meta-analysis or systematic review about tibial stems in primary TKA. The purpose of this study was to examine the indications for the usage of tibial stems in primary TKA, the types of stems used, and the associated outcomes, including revision rates and complications.

## Review

Methods

This systematic review was conducted as per the guidelines of the Preferred Reporting Items for Systematic Reviews and Meta-Analysis (PRISMA) [4]. Our systematic review involved an extensive search across electronic databases - PubMed, Embase, and Cochrane - from the inception of each database until January 31, 2024.

Our search strategy comprised specific terms applied to all searchable fields (title, keywords, and abstract), including "primary total knee replacement" OR "primary total knee arthroplasty" AND "tibial stems" OR "long stem" OR "stem extension". We selectively included studies published in English that presented clinical outcomes, radiographic outcomes, or complications associated with tibial stem utilization in primary total knee replacement surgeries.

Studies comparing stemmed and non-stemmed tibial baseplates, as well as studies focused solely on stemmed tibial baseplates without any comparison, were included. Studies without direct comparison groups are included to broaden the scope of evidence and capture valuable insights that may not be evident in comparative studies alone. These studies can provide data on outcomes such as survivorship, functional improvement, and complication rates, offering a more comprehensive understanding of the benefits and limitations of stemmed implants.

All studies involve human subjects, and provide full text, with no restrictions regarding publication date. Abstracts, commentaries, and other systematic reviews or meta-analyses, along with case reports, review articles, biomechanical studies, cadaveric studies, or those focusing on revision TKA, conversion of a unicompartmental knee arthroplasty (UKA), or joint replacement associated with oncologic resection, were excluded from consideration.

The risk of bias assessment was independently conducted by two investigators (PS and SC), with any disagreements resolved by a third investigator (AB). Observational cohort studies were evaluated using the Newcastle-Ottawa Scale [5], while randomized controlled trials were assessed using the Cochrane RoB 2 tool [6]. Each tool examined bias across several domains, assigning a rating of either "low risk", "high risk", or "unclear" for each domain. The overall risk of bias was determined by the highest level of risk identified in any single domain.

Results

**Figure 1:**
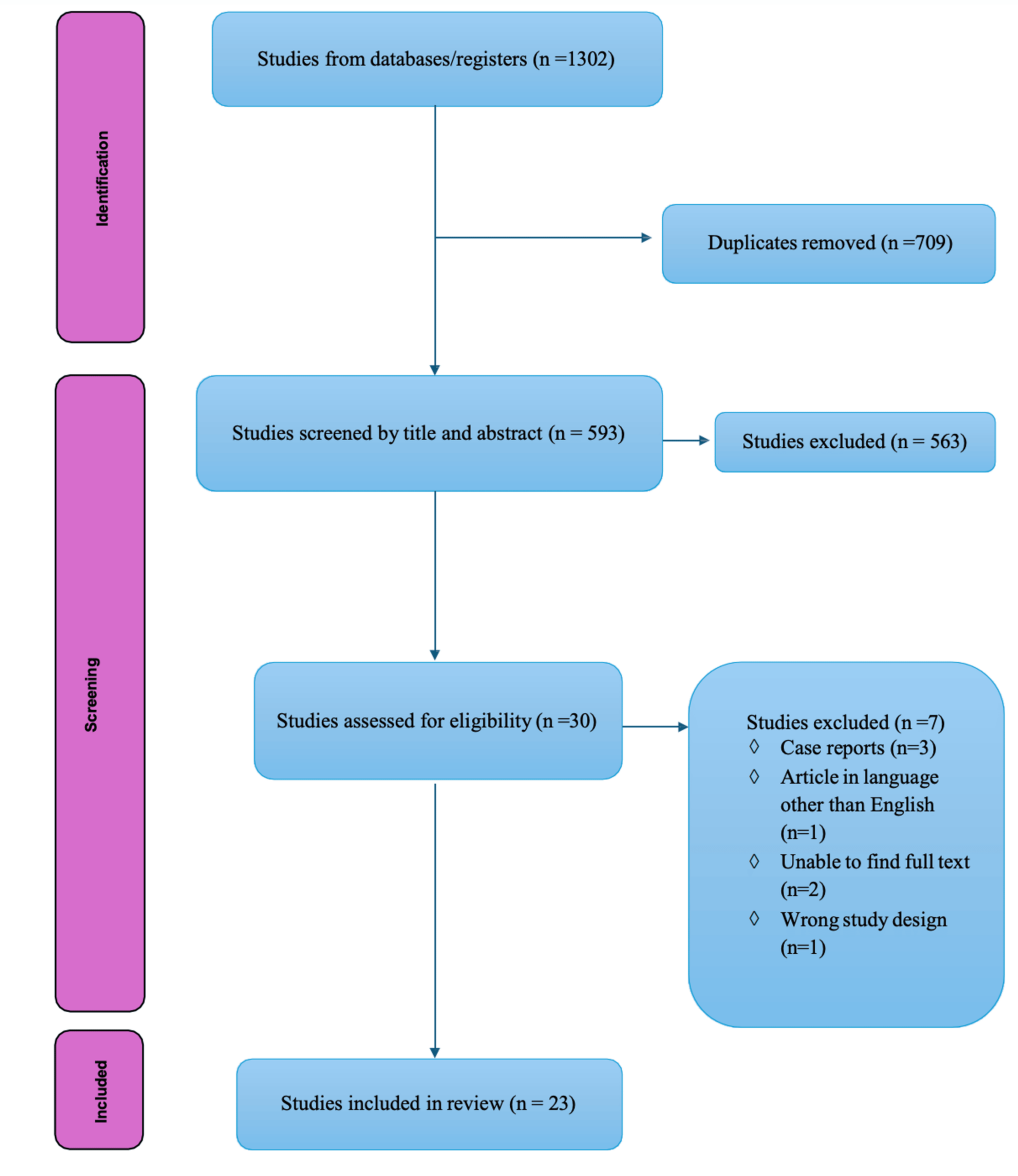
Prisma flowchart

Out of the 1,302 studies identified through literature searches, 23 met the selection criteria and were included in this systematic review. The screening process is detailed in the PRISMA flowchart (Figure 1). Collectively, these studies evaluated a total of 2,073 tibial stems, with extensions ranging in length from 30 to 145 mm. All eligible studies were published after 2013, and none disclosed any industry sponsorship or conflicts of interest. The majority of the studies were retrospective cohort studies, with a few being randomized controlled trials and prospective cohort studies as well. A comprehensive summary of the study characteristics is provided in Table 1.

**Table 1:** Summary of eligible studies

First author	Year	Country	Study design	Sample size (stemmed tibia)	Implant used	Tibial stem design	Tibial stem length (mm)
Parratte et al. [7]	2017	France	Randomised Controlled Trial	60	NexGen (Zimmer)	Tray cemented, stem press-fit	100
Elcock et al. [8]	2023	UK	Retrospective Cohort Study	54	Triathlon (Stryker)	Cemented	40
Mohammad et al. [9]	2023	Egypt	Randomised Controlled Trial	130	NexGen (Zimmer)	Tray cemented, stem press-fit	100
Steere et al. [10]	2018	USA	Retrospective Cohort Study	50	NexGen (Zimmer) or Persona (Zimmer)	Cemented	30
Garceau et al. [11]	2020	USA	Retrospective Cohort Study	162	Persona (Zimmer)	Cemented	30
Fournier et al. [12]	2020	France	Retrospective Cohort Study	35	Corin HLS	Cemented	30
Garceau et al. [13]	2022	USA	Retrospective Cohort Study	500	Persona (Zimmer)	Cemented	30
Elzohairy et al. [14]	2021	Egypt	Prospective comparative study	100	NexGen (Zimmer)	Cemented	100
Druel et al. [15]	2024	France	Retrospective Cohort Study	120	Persona (Zimmer) NexGen (Zimmer)	Tray-cemented, stem press-fit, or cemented	30 (cemented) or 100 (press-fit)
Angers-Goulet et al. [16]	2017	Canada	Prospective cohort study	91	Implants from Zimmer or Stryker	Cemented	30 or 50
Park et al. [17]	2018	Korea	Retrospective Cohort Study	99	NexGen (Zimmer)	Short cemented	Not stated
Fournier et al. [18]	2020	France	Retrospective Cohort Study	45	Corin HLS	Cemented	30
Hegde et al. [19]	2021	USA	Retrospective cohort study	67	Persona (Zimmer), Triathlon (Stryker), ATTUNE (DePuy) or SIGMA (DePuy)	Cemented	30 or 50
Samy et al. [20]	2022	Egypt	Retrospective Cohort Study	86	NexGen (Zimmer)	Tray cemented, stem press-fit	75 or 100
Cinotti et al. [21]	2022	Italy	Retrospective Cohort Study	8	SIGMA (DePuy) or Triathlon (Stryker)	Short cemented	Not stated
Barlow et al. [22]	2017	USA	Retrospective Cohort Study	95	Not stated	Not stated	Not stated
Mittal et al. [23]	2013	India	Retrospective Cohort Study	31	Not stated	Long uncemented	Not stated
Ebied et al. [24]	2018	Egypt	Prospective cohort study	18	NexGen LPS or LCCK (Zimmer)	Tray cemented, stem press-fit	145
Zhao et al. [25]	2022	China	Retrospective Cohort Study	81	PFC®SIGMA®TC3, (DePuy)	Long uncemented	Not stated
Tsukada et al. [26]	2013	Japan	Retrospective Cohort Study	33	Scorpio (Stryker)	Cemented	40 or 80
Hamai et al. [27]	2015	Japan	Retrospective Cohort Study	26	NexGen (Zimmer)	Tray cemented, stem press-fit	100
Durig et al. [28]	2014	USA	Retrospective Cohort Study	84	Profix (Smith&Nephew)	Short cemented	Not stated
Prudhon et al. [29]	2019	France	Matched Comparative Study	98	New Wave	Tray cemented, stem press-fit	100 or 120

All the studies included in this analysis had a follow-up period of at least 24 months. Table 2 provides a detailed presentation of the indications and outcomes of these studies.

**Table 2:** Indications and outcomes of tibial stems within the systematic review OA, osteoarthritis; BMI, body mass index; TKA, total knee arthroplasty; ROM, range of motion; AKSS, American Knee Society Score; KSS, Knee Society Score * Only range is available.

Study	Indication	Mean follow-up, months (range)	Result
Samy et al. [20]	Primary knee OA, Rheumatoid arthritis	24-108*	No difference in revision rate in short and intermediate follow-up periods
Park et al. [17]	Varus deformity >8 degrees	109 (range not available)	Short extension stem reduce the rate of tibial loosening
Garceau et al. [11]	Obese: BMI >40	36 (25-82)	Fully cemented stem reduce the risk of aseptic loosening
Parratte et al. [7]	Obese BMI >30	36 (24–48)	Some differences in patient-reported outcomes scores for pain and function favouring implants with stems, but the differences were small and unlikely to be clinically important.
Barlow et al. [22]	Post-traumatic arthritis, inflammatory arthritis, severe varus/valgus deformity >15 degrees	24 (range not available)	No difference between short stem vs long stem. No difference in revision rate between cemented vs uncemented stems
Elcock et al. [8]	Obese: BMI >40	58.7 (range not available)	No differences between standard keel and short-stemmed tibial baseplate
Fournier et al. [18]	Varus deformity >10 degrees	57 (27–171)	Extension stem leads to less loosening of the tibial component
Fournier et al. [12]	Obese BMI >30	52 (26 -108)	The use of tibial short stem extension was associated with a lower aseptic loosening rate
Mohammad et al. [9]	Obese BMI 30-40, Varus deformity <15	73.1 (61.1- 86.3)	Standard tibia TKA in obese patients with moderate varus in a 5-year follow-up period provided similar clinical and radiographic results to long-stemmed TKA
Hegde et al. [19]	Varus deformity >8	24 (range not available)	Stem use significantly reduced the risk of aseptic tibial component loosening requiring revision at a minimum 2-year follow-up
Garceau et al. [13]	Obese BMI >40	42 (33.6 – 75.5)	Short native tibial stem associated with early aseptic loosening
Steere et al. [10]	Obese BMI >35	32 (24-46)	No difference in the revision rate
Druel et al. [15]	Obese BMI >30	24 (range not available)	Use of a short cemented stem has better functional outcomes compared to a long stem and no stem
Durig et al. [28]	Osteoarthritis	81.5 (24-132)	Low incidence of tibial radiolucent lines and osteolysis on X-ray with tibial stem
Prudhon et al. [29]	Poor bone quality	96 (range not available)	Long stem improves alignment and fixation
Mittal et al. [23]	Tibia stress fracture	51 (24-96)	No complications
Zhao et al. [25]	Incompetent collateral ligaments, unbalanced flexion, and extension, bone defects	92.3 (range not available)	Similar clinical results and survivorship in both stemmed and no stem group
Hamai et al. [27]	Rheumatoid arthritis with bone defect	72 (24-134.3)	Effectively restore function. Significant improvements were observed postoperatively in maximum extension angle from −10 ° to −1 °, ROM from 101 ° to 115 °, and KSS (knee score/function score) from 35/18 to 90/63
Elzohairy et al. [14]	Obese BMI >35	91.2 (78–120)	Stemmed group has higher functional outcomes in AKSS
Angers-Goulet et al. [16]	Obese BMI >35, bone defect requiring bone graft, metal augment, or constraint	35.8 (24–85.4)	Short cemented stems result in good functional, clinical, and radiological outcomes for patients undergoing complex primary TKA
Tsukada et al. [26]	Medial tibial bone defect	36-72*	Stemmed tibia was not inferior to standard TKA
Cinotti et al. [21]	Varus deformity >12 degrees with bone defect	75.6( 60–98.4)	Comparable clinical outcome
Ebied et al. [24]	Tibia plateau fracture	72 (36–96)	Good outcome and midterm survival

The risk of bias was reported separately for observational and randomized studies, with results presented in Tables 3-4, respectively. There was a high risk of bias across most of the studies related to the representativeness of the exposed cohort. Studies limited to a single center or selected surgeon(s) were found to be at high risk of bias due to limited sample size. Additionally, the comparability of exposure and control groups was another source of observed bias. However, it should be noted that all eligible observational studies employed some form of independent or blinded assessment of revision, therefore reducing bias associated with the assessment of outcome. Of the two available randomized trials, one was determined to have a high risk of bias, while the other had a low risk of bias. In both studies, the randomization process was reported ambiguously. While missing data accounted for a small proportion of participants, it is important to note that the overall sample size in the randomized studies was generally small.

**Table 3:** Quality assessment of eligible observational studies Quality assessment performed using the Newcastle–Ottawa Scale for assessing the quality of non-randomized studies.

Study	Representativeness of the exposed cohort	Selection of the non-exposed cohort	Ascertainment of exposure	Demonstration that outcome of interest was not present at the start of the study	Comparability of cohorts on the basis of the design or analysis	Assessment of outcome	Was follow up long enough for outcomes to occur	Adequacy of follow-up of cohorts	Overall score
Fournier et al. [18]	Low	Low	Low	Low	Low	Low	High	High	6
Fournier et al. [12]	Low	Low	Low	Low	Low	Low	High	High	6
Garceau et al. [11]	High	Low	Low	Low	High	Low	Low	High	5
Garceau et al. [13]	High	Low	Low	Low	High	Low	High	Low	5
Steere et al. [10]	High	Low	Low	Low	High	Low	High	Unclear	4
Samy et al. [20]	High	Low	Low	Low	High	Low	High	Low	5
Park et al. [17]	High	Low	Low	Low	High	Low	High	Low	5
Barlow et al. [22]	High	Low	Low	Low	High	Low	High	Unclear	4
Elcock et al. [8]	Low	Low	Low	Low	Low	Low	High	High	6
Hegde et al. [19]	High	Low	Low	Low	High	Low	High	Unclear	4
Druel et al. [15]	High	Low	Low	Low	High	Low	High	Unclear	4
Durig et al. [28]	High	Low	Low	Low	High	Low	Low	High	5
Prudhon et al. [29]	High	Low	Low	Low	High	Low	High	Low	5
Mittal et al. [23]	High	Low	Low	Low	High	Low	Low	High	5
Zhao et al. [25]	High	Low	Low	Low	High	Low	High	Low	5
Hamai et al. [27]	Low	Low	Low	Low	Low	Low	High	High	6
Elzohairy et al. [14]	Low	Low	Low	Low	Low	Low	High	High	6
Angers-Goulet et al. [16]	High	Low	Low	Low	High	Low	Low	High	5
Tsukada et al. [26]	High	Low	Low	Low	High	Low	High	Low	5
Cinotti et al. [21]	High	Low	Low	Low	High	Low	High	Unclear	4
Ebied et al. [24]	High	Low	Low	Low	High	Low	High	Low	5

**Table 4:** Quality assessment of eligible randomiced controlled trials Quality assessment performed using the Revised Cochrane Risk-of-Bias Tool for Randomized Trials (RoB 2).

Study	Risk of bias arising from the randomization process	Risk of bias due to deviations from the intended interventions (effect of assignment to intervention)	Missing outcome data	Risk of bias in the measurement of the outcome	Risk of bias in the selection of the reported result	Overall risk of bias
Parratte et al. [7]	Unclear	Low	Low	Low	Unclear	Low
Mohammad et al. [9]	Unclear	High	Unclear	Low	High	High

The primary indication for using tibial stems was in obese patients, with varying body mass index (BMI) thresholds reported across different studies. While the BMI criteria differed among studies, all consistently applied the WHO definition of obesity (BMI ≥ 30 kg/m²). Most studies considered a BMI over 30 as a threshold, though some focused on higher levels, such as a BMI over 35 kg/m² and morbid obesity with a BMI of 40 kg/m². Among these, four studies found no discernible difference in outcomes and revision rates between stemmed and non-stemmed tibial components [7-10]. Paratte et al. noted small differences in patient-reported outcomes favoring stemmed implants but deemed these differences clinically insignificant [7]. Elcock et al. similarly found no significant differences in joint-specific function, quality of life, or complication rates between stemmed and non-stemmed implants in obese patients [8]. Mohammad et al. reported comparable clinical and radiographic outcomes between standard and long-stemmed tibial implants in obese patients with moderate varus [9].

Conversely, seven studies reported a lower revision rate for aseptic loosening of the stemmed tibial component, along with improved functional and radiological outcomes [11-14]. Garceau et al. observed increased rates of tibial aseptic loosening in morbidly obese patients with standard stems compared to those with stemmed tibias [11]. Fournier et al. found that using tibial short stem extensions was associated with lower rates of aseptic loosening in obese patients [12]. Elzohairy et al. highlighted significant instability in the non-stemmed group of obese patients due to loosening [14]. Studies evaluating functional outcomes using the Knee Society Score (KSS) indicated better outcomes with short cemented stems compared to no stem in obese patients [14-16].

The use of tibial stems to address varus deformity emerged as another significant indication. Studies examined various thresholds for femoral tibial angles (FTA) and hip-knee ankle (HKA) angles. Studies consistently demonstrated lower rates of aseptic loosening in tibial components for patients with severe varus deformity [17-19]. Park et al. found a notably reduced incidence of loosening on the tibial side when using short extension stems in patients with severe preoperative varus deformity [17]. Hegde et al. supported this by demonstrating a significant risk reduction in aseptic tibial component loosening with stem utilization in severe preoperative varus cases [19]. Fournier et al. reported no significant differences in surgical outcomes but noted a higher occurrence of tibial loosening in severe varus patients with standard stems compared to those with extension stems [18]. Samy et al. highlighted statistically significant improvements in the KSS and Knee Society Functional Score (KSFS) among patients with severe varus malalignment [20]. However, other studies, such as Cinotti et al., suggested comparable outcomes in severe varus patients treated with standard TKA [21]. Barlow et al. reported similar revision rates between stemmed and conventional TKA in severe varus patients [22].

Another notable indication involved patients with osteoarthritis, who also had tibial stress or plateau fractures. For these patients, long uncemented stems were used, leading to favorable outcomes and minimal complications [23,24].

In various studies, tibial bone defects necessitating metal augments were treated with tibial stems, yielding positive functional, clinical, and radiological outcomes [25,26]. This treatment was also suitable for patients with rheumatoid and inflammatory arthritis with bone defects. As noted by Hamai et al., the use of metal block augmentation and stem extension in primary TKA effectively restored function in patients with severe knee joint destruction due to rheumatoid arthritis [27].

Additional indications included post-traumatic arthritis, unbalanced flexion and extension gaps, and poor bone quality, all of which demonstrated positive outcomes with proper alignment and fixation [25,28,29].

Various stem lengths were employed in the studies, ranging from short stems at 30 mm to long stems spanning from 100 to 145 mm. Short stems were universally cemented, while long stems were either cemented or uncemented. The classification of tibial stems into "short" and "long" varies across studies and clinical practices, with no universally standardized lengths. The choice between cemented and press-fitted tibial long stems depends on bone quality, patient age, and surgical indication, with cemented stems preferred for poor bone stock and press-fitted stems for younger patients with good bone stock.

Complications associated with tibial stems were observed (Table 5). These include one stem-related tibial fracture with the long stem, four cases of end-of-stem pain with long stems, three instances of deep vein thrombosis (DVT), one pulmonary embolism, two cases of transient peroneal nerve palsy, three incidents of skin necrosis, and five infections, one of which necessitated debridement, antibiotics, and implant retention (DAIR) procedure. Cortical sclerosis was noted at the stem tip in one study but remained asymptomatic [18]. None of the studies reported an increase in operative time, tourniquet time, or bleeding.

**Table 5:** Complication of tibial stems in primary total knee arthroplasty

	Mohammad et al. [9]	Zhao et al. [25]	Angers-Goulet et al. [16]	Tsukada et al. [26]	Ebied et al. [24]
Superficial infection	-	2 of 81	-	-	-
Deep infection	-	1 of 81	2 of 91	-	1 of 18
Skin necrosis	-	-	1 of 91	1 of 33	-
Fracture	1 of 130	-	-	-	-
Deep vein thrombosis	-	-	3 of 91	-	-
Pulmonary embolism	-	-	1 of 91	-	-
End of stem pain	-	4 of 81	-	-	-
Transient peroneal palsy	-	-	2 of 91	1 of 33	-
Total	1 of 130	7 of 81	9 of 91	2 of 33	1 of 18

Discussion

The use of stems in primary TKA remains a topic without a clear consensus in the literature, although evidence suggests potential benefits in specific clinical scenarios.

Stemmed tibial components are commonly used to enhance primary tibial survival and improve component rigidity against bending forces. Finite element analysis has shown that stem extensions also decrease stress at the cement-device interface, thereby reducing micromotion and enhancing implant stability [30]. However, these advantages are accompanied by drawbacks such as stress shielding, the potential for complex revisions, and the risk of stem-related complications such as periprosthetic fractures and stem-end pain.

The use of shorter stem extensions may mitigate these disadvantages by minimizing bone removal, and simplified revision procedures, as well as mitigating issues related to proximal tibial meta-diaphyseal stem conflict and reducing the incidence of end-of-stem pain compared to press-fit stems [25].

Short cemented stems have garnered particular interest in complex primary TKAs, especially among obese patients who are at higher risk of revision surgery due to aseptic tibial loosening [31]. Short cemented stems offer several advantages for patients undergoing primary TKA. Their compact design allows for greater flexibility in positioning along the medial/lateral and anterior/posterior axes, which enhances component alignment in the coronal plane. Additionally, they help conserve bone stock and promote optimal load distribution. Within this analysis, shorter stems are not associated with any stem-related complications in the patient cohorts studied. Furthermore, comparative studies have shown that short stems yield superior functional outcomes compared to long stems, as indicated by the KSS and IKS [15].

When considering long stems for obese patients, the advantages are not significant. Parratte et al. conducted a prospective study involving 120 patients with BMI ≥30 kg/m^2^ who were randomly assigned to receive either a 100 mm length tibial stem extension or standard tibial components [7]. The study concluded after two years, during which the authors found no differences in mechanical complications. They cautioned against the routine use of long stems, citing increased surgical complexity and revision challenges associated with these implants.

However, long stems have their own benefits, particularly in cases involving osteoarthritis with stress fractures and tibial plateau fractures. Nonoperative treatment of stress fractures associated with osteoarthritis has been discussed in the literature. However, this approach can lead to knee stiffness following immobilization. Additionally, failure to address the underlying deformity increases the risk of refracture and non-union. TKA with long tibial stems offers an effective solution to these challenges, providing stability and reducing the likelihood of further complications. Similarly, elderly patients with tibial plateau fractures often face fixation difficulties due to osteoporosis and metaphyseal comminution. Mobilizing these patients with partial weight-bearing is challenging as early weight-bearing can compromise fixation integrity, while immobilization leads to muscle wasting and joint stiffness. In such cases, TKA with metal augments supporting the tibial tray proves beneficial, particularly for significant bone defects at the articular surface. The use of intramedullary diaphyseal stem contact promotes primary stability and supports early weight-bearing, ensuring the stability of the tibial trays.

In addition to tibial stress or plateau fractures associated with underlying osteoarthritis, long tibial stems are also employed in treating inflammatory arthritis. Even with the recent advancements in rheumatoid arthritis (RA) pharmacotherapy, surgeons performing TKA continue to face challenges with severe cases of RA characterized by large bone defects, severe deformity, and other advanced forms of knee joint destruction. In cases where bone defects are present, strategies such as bone graft transplantation or the use of metal augmentation with long stem extension are employed.

Consideration should be also given to utilizing stems in patients undergoing complex primary TKA requiring constraint. While the use of constrained prostheses in primary TKA is uncommon, it may be warranted for patients with severe deformities, incompetent collateral ligaments, severe flexion contracture, and difficulty achieving knee balance in flexion and extension. In a recent retrospective study, Ruel et al. observed significant femoral component loosening with stemless constrained knee components [32]. Similarly, Kajetanek et al. reported a higher rate of revision for aseptic loosening in their retrospective series comparing stemless versus standard stem TKA [33]. These findings underscore the importance of using a stem in complex primary TKAs that require constraint, aiming to mitigate early aseptic loosening issues.

Osan et al. analyzed data from the Australian joint registry, comparing revision rates and reasons for revision in primary TKA performed for osteoarthritis, both with and without tibial stem extensions [34]. Their findings showed that patients who underwent primary TKA with stemmed tibial components had lower rates of all-cause revisions after 1.5 years and lower tibial component-only revisions at all time points. However, between three months and 1.5 years post-surgery, stemmed components were more frequently revised due to infection.

While this study employed systematic and rigorous methods, several limitations must be acknowledged. To begin with, the average follow-up period of 24 months across the included studies is relatively short, considering the expected lifespan of a TKA, which may limit the relevance of the findings over the long term. Additionally, the retrospective design of most studies may have introduced bias. This review included only two randomized controlled trials, the gold standard for minimizing bias, and they found no significant difference in overall outcomes between stemmed and non-stemmed implants. Several confounding factors could also impact the results, including variations in tibial stem length across different implants, cementation techniques, types of cement and polyethylene, and alignment before and after TKA, as well as differences in bone quality-all recognized as risk factors for aseptic revision. Furthermore, inconsistencies in the BMI thresholds for obesity and the degree of varus deformity add another layer of complexity. Given the probable positive correlation between higher BMI, greater deformity, and increased rates of aseptic revision, the variability in these criteria may contribute to heterogeneity in the outcomes. Additional sources of heterogeneity include differences in patient populations, associated pathologies, osteoarthritis grading, and the outcome measures used. Moreover, unrecognized factors, such as the involvement of multiple surgeons, variations in rehabilitation protocols, and observer bias, could have influenced the study results. The lack of sufficiently homogeneous comparative studies prevented the possibility of conducting a meta-analysis.

## Conclusions

The most significant finding from this systematic review is that the use of tibial stems did not compromise the survival rates of primary TKA, which is a highly positive outcome. In particular, the use of short cemented stems in complex primary TKA cases demonstrated favorable functional, clinical, and radiological outcomes. This highlights their appropriateness, especially for obese patients and those with severe deformities, where maintaining the integrity and longevity of the implant is crucial.

Short cemented stems offer several advantages, including the effective preservation of bone stock, optimized load transmission, and a reduction in complications such as stem-related fractures and end-of-stem pain. Moreover, they are more cost-effective compared to longer stems, making them a practical choice in both clinical and economic terms. Looking ahead, it is essential to conduct further research and long-term follow-up studies to establish standardized guidelines for the optimal use of tibial stems in primary TKA. Such efforts will be vital in ensuring improved clinical outcomes and enhancing patient satisfaction across a variety of clinical scenarios.
